# Impact of National HIV and AIDS Communication Campaigns in South Africa to Reduce HIV Risk Behaviour

**DOI:** 10.1100/2012/384608

**Published:** 2012-11-08

**Authors:** Karl Peltzer, Warren Parker, Musawenkosi Mabaso, Elias Makonko, Khangelani Zuma, Shandir Ramlagan

**Affiliations:** ^1^HIV/AIDS, STIs and TB (HAST), Human Sciences Research Council, Private Bag X41, Pretoria 0001, South Africa; ^2^Department of Psychology, University of Limpopo, Turfloop Campus, Private Bag X1106, Sovenga 0727, South Africa; ^3^Centre for AIDS Development, Research and Evaluation, P.O. Box 30829, Braamfontein 2017, South Africa

## Abstract

In South Africa social and behavioural communication interventions are a critical component of HIV/AIDS prevention, and numerous communication campaigns have been implemented intensively across the country through government initiatives and nongovernmental organisations over the past decade. The aim of this paper is to assess the reach of HIV and AIDS communication campaigns in conjunction with contributions to knowledge, attitudes, and HIV risk behaviours in the general population in South Africa. The sample included in this nationally representative cross-sectional survey was 13234 people aged 15–55 years. Overall, the study found that there was high exposure to 18 different HIV communication programmes (median 6 programmes and 14 programmes more than 30%) across different age groups. Most programmes were more often seen or heard by young people aged between 15 and 24 years. In multivariate analysis, greater exposure to HIV mass communication programmes was associated with greater HIV knowledge, condom use at last sex, having tested for HIV in the past 12 months, and less stigmatizing attitude toward PLWHA.

## 1. Introduction

Population-wide mass media can use the combination of television, radio and print. This includes television and radio, episodes as well as inserts in key newspapers during each year of intervention, and the campaigns are repeated annually. Bertrand et al. [1] examined the effectiveness of 24 mass media interventions on changing HIV-related knowledge, attitudes- and behaviours in low- and middle-income countries. The most frequently reported outcomes were condom use (17 studies) and knowledge of modes of HIV transmission (15), followed by reduction in high-risk sexual behaviour (8), perceived risk of contracting HIV/AIDS (6), interpersonal communication about AIDS or condom use (6), self-efficacy to negotiate condom use (4), and abstaining from sexual relations (3). The studies yielded mixed results, and where statistically significant, the effect size was small to moderate (in some cases as low as 1-2% point increase). On two of the seven outcomes, at least half of the studies did show a positive impact of the mass media in the improvement of knowledge of HIV transmission and reduction of high-risk sexual behaviour [1]. In a more recent review, Noar et al. [2] evaluated 34 distinct HIV/AIDS campaign efforts conducted in 23 countries and found among the 30% of studies with stronger outcome evaluation designs, that the vast majority of the well-controlled studies demonstrated effects on knowledge (increased AIDS knowledge), behaviour (increased condom use; reductions in numbers of sexual partners; were more likely to get tested for HIV), or behavioural intentions (increased condom use intentions). In addition, several studies [3–5] found that exposure to HIV-related communication on the media was significantly related to less stigmatizing attitude toward people living with HIV and AIDS (PLWHA).

In South Africa social and behavioral communication interventions are a critical component of HIV/AIDS prevention [6]. Numerous communication campaigns have been implemented intensively across the country through government initiatives and nongovernmental organisations over the past decade [6–8]. The purpose is to help combat the spread of HIV and AIDS by improving knowledge about modes of transmission, risk perceptions, changing sexual behaviours, questioning potential harmful social norms, and promoting resources and services that support prevention [6, 9]. 

Campaigns utilise radio, television, and other outlets to address prevention of HIV/AIDS by conveying messages through interpersonal, community, and national channels [7, 8]. Four major national-level HIV and AIDS communication programmes using mass media and other communication channels include Khomani, meaning “caring together;” LoveLife, which focuses mainly on teenagers, Soul City and Soul Buddyz which address adults and children, respectively, and the 46664 campaign which aims to promote HIV prevention through a series of events and activities linked to the ethos and values of Nelson Mandela. This latter campaign is conducted nationally, but also extends globally [6, 8].

National HIV/AIDS communication surveys demonstrated impacts in the improvement of knowledge and awareness and have illustrated outcomes related to HIV prevention [7–9]. An evaluation of Soul City in South Africa illustrates the mediation of community change as a product of communication programming [10]. In a national communication survey in 2009, Johnson et al. [6] found that HIV communication programmes as a whole brought about positive changes in condom use and HIV testing but did not impact on reduction of multiple sexual partners. In an evaluation of a youth programme (loveLife) (combining multimedia and community outreach and support programmes) in South Africa, Pettifor et al. [11] found that the participation in loveLife reduced chances of being infected with HIV, and youth were more likely to report condom use at last sex and used condoms more consistently.

The aim of this paper is to assess the reach of these communication campaigns in conjunction with contributions to knowledge, attitudes, and HIV risk behaviours in the general population in South Africa.

## 2. Methods

### 2.1. Sample and Procedure

The survey targeted all persons over 2 years of age living in South Africa and residing in homes (i.e., excluding individuals living in educational institutions, old-age homes, hospitals, and uniformed service barracks but including those living in hostels). A multistage cluster stratified sample stratified by province, settlement geography (geotype), and predominant population group in each area was used. A systematic sample of 15 households was drawn from each of 1 000 census enumeration areas (EAs). In each household, one person was randomly selected in each of the four mutually exclusive age groups (under 2 years; 2–14 years; 15–24 years; 25+ years). Sociodemographic and behavioural information was collected with questionnaires administered by trained field workers (more details on the methodology are described by Shisana et al., [8]). The age group selected for analysis in this paper was 13234 people aged 15–55 years. This age range was chosen because of greater HIV risk.

Ethical approval for conducting the study was obtained from the Human Sciences Research Council's Ethics Committee (Application number REC5/24/04). Informed consent was obtained for agreeing to participate in the interview. Registered professional nurses were trained to conduct interviews.

### 2.2. Measure

A structured questionnaire was used to determine the reach of national mass media communication campaigns that promote HIV/AIDS education in South Africa. Respondents were asked questions related to mass media reach as well as their knowledge of campaigns and perceptions of HIV/AIDS communication in general. The sections of the questionnaire analysed here included access to media channels and exposure to 18 different HIV mass communication programmes in the past 12 months in South Africa. Impact of the HIV mass communication programmes was measured in terms of HIV knowledge (16 items, Cronbach's alpha of 0.72), sexual risk behaviour (multiple sexual partners, condom use, HIV testing), and HIV stigma attitudes (5 items, Cronbach's alpha of 0.54).

## 3. Data Analysis

To create a joint index of HIV mass communication exposure, each programme was coded 0/1 for recall and then summed to create scale from 0 to 18. By taking the median of 6 as a cut-off point, this scale was dichotomised as low or high exposure, and it was further grouped into three groups of 1 = 0–3 exposures, 4–8, and 9 or more exposures to mass communication programmes. Other dependent variables included *HIV knowledge*, which was dichotomised into 1 = 14–18 correct responses and 0 = 0–13 correct responses and *HIV/AIDS stigma*, which was dichotomised into 1 = 2–5 scores and 0 = 0–1 scores. Accounting for complex sampling design, a weighted analysis of the outcome of interest was carried out for the main reporting domains. Weighted data were analyzed using STATA 10.0 software. STATA suite of survey commands was used to obtain estimates with 95% confidence intervals that took into account the survey design. Adjusted odds ratios (ORs) are reported to indicate the strength and direction of association. A *P* value less than 5% is used to indicate statistical significance. 

## 4. Results

### 4.1. Access to Media

Radio and television are the most popular mass media consumed by people in South Africa, with 81.7% of people watching TV and 83.6% listening to the radio. While 66.9% and 60.7% of people reported reading a newspaper and a magazine, respectively, only 22.4% and 12.9% reported reading a newspaper and a magazine, respectively, every day. The internet was the least accessed channel, with 17.7% of the population accessing it (see Table 1).

### 4.2. Exposure to HIV Mass Communication Programmes

Exposure is defined as have heard or seen at least one of any HIV mass communication programme such as billboard, radio, TV, or other components in the past 12 months prior to the survey interview. Overall, a high exposure to 18 different HIV communication programmes (median 6 programmes and 14 more than 30%) across different age groups was found. Most programmes were more often seen or heard by youth (15–24 years), and this is in addition to programmes specifically designed for youth such as loveLife and Soul Buddyz (see Table 2).

The median exposure to HIV mass communication among participants was 6 programmes, IQR 3–9. In the multivariate analysis, younger age, higher education, urban formal residence, and the African population group were associated with high HIV mass communication exposure (see Table 3).

### 4.3. Association between HIV Mass Communication Programmes and HIV Risk Behaviour

In multivariate analysis, greater exposure to HIV mass communication programmes was associated with greater HIV knowledge, condom use at last sex, having been tested for HIV in the past 12 months, and less stigmatizing attitude toward PLWHA (see Table 4). 

## 5. Discussion

Overall the study found that there was high exposure to the 18 different HIV communication programmes among the general population, especially among youth. Higher HIV mass communication exposure was in this study associated with improved HIV knowledge and lowered HIV/AIDS stigma, as found in previous studies [3–5, 12–15]. Further, higher HIV mass communication exposure was in this study, in agreement with other studies, associated with the reduction of HIV risk behaviour (e.g., condom use at last sex [6, 11, 15–19] and having had an HIV test in the past 12 months) [20–23]. The study did not find any association between higher HIV mass communication exposure and reduction of the number of sexual partners, as found in some other studies [18, 24, 25]. Trend data from population-based surveys in South Africa seem to confirm an increase in condom use, in particular among young people, but not a reduction in the number of sexual partners. Overall, the current study adds to the growing literature suggesting that mass media campaigns can be effective in changing HIV risk behaviour and attitudes, at least over the short term [2, 19]. This finding confirms the development of a shift in the purpose of campaigns, from simply aiming to raise awareness about HIV and AIDS to attempting to impact safer sexual behaviours [2].

## 6. Study Limitations

The study design used was a weak (i.e., preexperimental) outcome evaluation design; more rigorous quasi-experimental designs are needed [2] including multiple assessments [19]. Exposure to communication campaigns relied on self-reporting, and it was not possible to measure the intensity of exposure, which should be included in further studies. The communication campaigns were not the only potential exposure to HIV and AIDS information among respondents, and direct and exclusive contributions to change cannot therefore be claimed. Further, the study found a low reliability of the HIV/AIDS stigma index used; therefore, if the stigma scale had been used as a scale, there would have been some limitation in validity of results. Finally, there are several biomedical and structural issues which the mass media could well have been instrumental for; their utilization in sub-Saharan Africa seems to be limited to behavioural interventions [26].

## 7. Conclusions

Exposure to communication campaigns in South Africa is associated with a range of outcomes relevant to addressing HIV and AIDS in relation to attitudinal aspects of the disease—particularly stigma reduction—and for HIV prevention. Communication campaigns thus provide a broad backdrop of support to HIV prevention activities and are likely to be strengthened via coherent and systematic prevention activities conducted at the community level. 

## Figures and Tables

**Table 1:** Percentage of people accessing various media channels.

Media channels	Frequency of accessing various media channels			
	Never	Once a week	2–6 times a week	Every day of the week
Listen to radio	16.4	9.9	16.5	57.1
Watch television	18.3	5.5	10.3	65.9
Read a magazine	39.3	29.0	18.8	12.9
Read a newspaper	33.1	26.6	18.0	22.4
Use the internet	82.3	4.9	4.6	8.2

**Table 2:** HIV mass media communication exposure in the past 12 months by age group expressed as percentages.

Mass media programme	Age 15–24 *N* = 5344	Age 25–35 *N* = 2644	Age 36–55 *N* = 5246	Age 15–55 *N* = 13234
	%	%	%	*N* (%)
(1) Watched Soul City on television	62.0	58.9	49.5	5718 (56.6)
(2) Listened to Soul City on the radio	36.6	37.1	34.7	3374 (36.0)
(3) Watched Beat It—Siyanqoba on television	26.7	27.6	20.4	2264 (24.7)
(4) Watched Masupatsela on television	12.2	13.0	10.5	1122 (11.8)
(5) Listened to a talk show about AIDS on television	56.8	57.9	52.0	6015 (55.4)
(6) Listened to a talk show about AIDS on the radio	48.5	50.3	47.6	4891 (48.7)
(7) Seen a loveLife advert on television	66.5	60.4	49.0	6381 (58.4)
(8) Heard a loveLife advert on radio	51.9	52.3	40.8	4828 (48.0)
(9) Seen a loveLife billboard	61.2	58.9	46.8	5892 (55.3)
(10) Read UnCut (loveLife youth magazine)	32.7	21.2	14.5	2486 (22.7)
(11) Seen a Khomanani advert on television	59.3	56.4	48.0	5455 (54.4)
(12) Heard a Khomanani advert on radio	44.5	45.1	38.7	4026 (42.5)
(13) Heard about New Start HIV testing centres	21.2	23.7	20.2	2515 (21.6)
(14) Watched Soul Buddyz on television	60.6	51.8	39.4	5338 (50.3)
(15) Read Soul City Booklet	44.9	34.4	25.0	3638 (34.5)
(16) Watched Buddyz on the move on television	44.7	35.8	24.4	3493 (34.7)
(17) Heard about the 46664	58.8	58.8	52.1	6279 (56.4)
(18) Heard about the play “Khululeka/It's in our hands.”	26.1	30.0	24.9	2561 (26.8)

**Table 3:** Association of demographic characteristics and overall exposure to HIV communication campaigns (*N* = 13234).

Demographic characteristics	*N* (%)	AOR (95% CI)	*P* value*
Age			
15–24	5344 (35.1)	1.00	
26–35	2644 (29.3)	0.77 (0.65–0.90)	0.001
36–55	5246 (35.6)	0.71 (0.61–0.82)	<0.001
Sex			
Women	7518 (52.0)	1.00	
Men	5716 (48.0)	1.05 (0.92–1.20)	0.481
Education			
Grade 0–7	2266 (19.8)	1.00	
Grade 8–11	5288 (42.8)	3.14 (2.61–3.78)	<0.001
Grade 12 or more	4775 (37.4)	5.34 (4.36–6.54)	<0.001
Geolocality			
Urban formal	8290 (54.2)	1.00	
Urban informal	1577 (10.2)	0.55 (0.42–0.73)	<0.001
Rural	3367 (35.6)	0.34 (0.27–0.42)	<0.001
Population group			
White	1509 (9.2)	1.00	
Black African	7750 (78.5)	0.10 (0.07–0.13)	<0.001
Coloured	2512 (9.6)	0.70 (0.55–0.89)	0.003
Indian or Asian	1428 (2.6)	0.32 (0.19–0.54)	<0.001

Adjusted odds ratio: AOR. *Significance tested using logistic regression at *P* value <0.05.

**Table 4:** Association between HIV communication campaigns and outcome variables.

Outcome variables	HIV knowledge (63.1%)	Condom use at last sex (61.2%)	Two or more sexual partner in the past 12 months (9.8%)	Tested for HIV in the past 12 months (24.7%)	HIV stigma attitudes (32.8%)
	AOR (95% CI)^1^	AOR (95% CI)^1^	AOR (95% CI)^1^	AOR (95% CI)^1^	AOR (95% CI)^1^
Age					
15–24	1.00	1.00	1.00	1.00	1.00
26–35	1.17 (0.98–1.40)	0.39 (0.31–0.48)***	0.59 (0.43–0.81)***	1.75 (1.47–2.09)***	0.95 (0.80–1.14)
36–55	1.19 (1.02–1.39)*	0.34 (0.27–0.43)***	0.28 (0.20–0.39)***	1.38 (1.16–1.64)***	1.03 (0.87–1.22)
Sex					
Women	1.00	1.00	1.00	1.00	1.00
Men	0.80 (0.70–0.92)**	1.28 (1.07–1.53)**	5.27 (4.62–8.50)***	0.64 (0.55–0.74)***	1.27 (1.12–1.44)***
Education					
Grade 0–7	1.00	1.00	1.00	1.00	1.00
Grade 8–11	1.63 (1.38–1.94)***	0.99 (0.75–1.31)	1.10 (0.73–1.67)	0.95 (0.77–1.16)	0.67 (0.56–0.80)***
Grade 12 or more	3.29 (2.65–4.08)***	0.82 (0.61–1.11)	1.04 (0.69–1.57)	1.32 (1.05–1.67)*	0.44 (0.36–0.54)***
Geolocality					
Urban formal	1.00	1.00	1.00	1.00	1.00
Urban informal	0.99 (0.81–1.21)	0.89 (0.66–1.18)	0.75 (0.49–1.16)	0.99 (0.79–1.25)	0.92 (0.71–1.20)
Rural	0.86 (0.72–1.02)	0.92 (0.73–1.16)	0.88 (0.63–1.23)	0.96 (0.79–1.16)	1.65 (1.35–2.01)***
Population group					
Black African	1.00	1.00	1.00	1.00	1.00
White	2.08 (1.53–2.84)***	0.22 (0.16–0.30)***	0.58 (0.34–1.00)*	0.85 (0.63–1.15)	1.58 (1.22–2.05)***
Coloured	0.76 (0.64–0.90)**	0.28 (0.22–0.36)***	0.77 (0.52–1.15)	0.82 (0.66–1.01)	2.41 (1.99–2.93)***
Indian or Asian	1.25 (0.78–2.00)	0.22 (0.15–0.34)***	0.10 (0.05–0.20)***	0.52 (0.38–0.71)***	2.19 (1.53–3.14)***
HIV communication Campaigns					
0–3	1.00	1.00	1.00	1.00	1.00
4–8	1.50 (1.26–1.79)***	1.30 (1.03–1.64)*	1.00 (0.70–1.41)	1.03 (0.86–1.24)	0.71 (0.60–0.84)***
9 or more	1.99 (1.67–2.36)***	1.28 (1.00–1.62)*	0.93 (0.65–1.33)	1.45 (1.19–1.78)***	0.41 (0.34–0.49)***

Adjusted odds ratio: AOR. ^1^Adjusted by media access (radio, TV, newspaper, magazine, and Internet). Significance tested using logistic regression at ***P < 0.001; **P < 0.01; *P < 0.05.
